# Effects of Biochar on the Growth and Development of Tomato Seedlings and on the Response of Tomato Plants to the Infection of Systemic Viral Agents

**DOI:** 10.3389/fmicb.2022.862075

**Published:** 2022-05-09

**Authors:** Marta Luigi, Ariana Manglli, Immacolata Dragone, Maria Grazia Antonelli, Mario Contarini, Stefano Speranza, Sabrina Bertin, Antonio Tiberini, Andrea Gentili, Leonardo Varvaro, Laura Tomassoli, Francesco Faggioli

**Affiliations:** ^1^Council for Agricultural Research and Economics-Research Centre for Plant Protection and Certification (CREA-DC), Rome, Italy; ^2^Department of Agriculture and Forest Sciences, University of Tuscia, Viterbo, Italy

**Keywords:** potato spindle tuber viroid, tomato spotted wilt virus, biochar, tomato, systemic infection

## Abstract

Biochar is a rich carbon product obtained by pyrolysis of biomass under a limited supply of oxygen. It is composed mainly of aromatic molecules, but its agronomic value is hard to evaluate and difficult to predict due to its great variable characteristics depending on the type of starting biomass and the conditions of pyrolysis. Anyway, it could be used as soil amendment because it increases the soil fertility of acidic soils, increases the agricultural productivity, and seems to provide protection against some foliar and soilborne diseases. In this study, the effects of biochar, obtained from olive pruning, have been evaluated on tomato seedlings growth and on their response to systemic agents' infection alone or added with beneficial microorganisms (*Bacillus* spp. and *Trichoderma* spp.). First, experimental data showed that biochar seems to promote the development of the tomato seedlings, especially at concentrations ranging from 1 to 20% (w/w with peat) without showing any antimicrobial effects on the beneficial soil bacteria at the tomato rhizosphere level and even improving their growth. Thus, those concentrations were used in growing tomato plants experimentally infected with tomato spotted wilt virus (TSWV) and potato spindle tuber viroid (PSTVd). The biochar effect was estimated by evaluating three parameters, namely, symptom expression, number of infected plants, and pathogen quantification, using RT-qPCR technique and −ΔΔCt analysis. Biochar at 10–15% and when added with *Trichoderma* spp. showed that it reduces the replication of PSTVd and the expression of symptoms even if it was not able to block the start of infection. The results obtained on TSWV-infected plants suggested that biochar could contribute to reducing both infection rate and virus replication. For systemic viral agents, such as PSTVd and TSWV, there are no curative control methods, and therefore, the use of prevention means, as can be assumed the use biochar, for example, in the nursery specialized in horticultural crops, can be of great help. These results can be an encouraging starting point to introduce complex biochar formulates among the sustainable managing strategies of plant systemic diseases.

## Introduction

Nowadays, biomass conversion represents a new approach to obtain renewable energy in several fields, such as urban waste recycling, biofuel production, and industrial processes. The agricultural productions also benefit from biomass-derived amendments for sustaining the soil fertility. Biochar is a solid and carbonaceous amendment obtained from various materials by pyrolysis, a heating process under a limited supply of oxygen (Laird et al., 2009), and it has been described as an “anthropogenically produced black carbon (BC) material” (Lorenz and Lal, 2018). In the twenty-first century, the use of biochar has seen a renewed interest because of its longevity in the soil, estimated between hundreds and thousands of years or more, and its role as a carbon removal from the atmosphere, sequestering CO_2_, with beneficial effects in mitigating the climate changes (Frenkel et al., 2017). Biochar is composed mainly of aromatic molecules with base cations that form bridges between the organic particles of the soil. These structures improve the soil pH (Yamato et al., 2006), as well as the retention of both the soil water and nutrients (Bronick and Lal, 2005; Chan et al., 2007, 2008; Rajkovich et al., 2012). In this way, biochar promotes the transformation and turnover of nutritional elements (Pietikäinen et al., 2000) and contributes to expand beneficial microbial population like rhizobacteria and fungi (Graber et al., 2010) and neutralize phytotoxic molecules (Wardle et al., 1998). The biochar effects on the soil biomass composition often result in enhanced plant fitness and productivity, as already observed for several crops such as wheat, maize, cucumber, bean, tomato, strawberry, and sweet pepper (Graber et al., 2010; Harel et al., 2012; Cornelissen et al., 2013; Jaiswal et al., 2014, 2015; De Tender et al., 2016). In a few cases, no effects, or even unwanted effects, were described (Jeffery et al., 2011; Kammann et al., 2015; Haider et al., 2016; Shackley et al., 2016), showing that the agronomic value of biochar can be variable. This is mainly due to the fact that both structure and biological activity of biochar can be affected by several factors, including the type of starting biomass and the conditions of pyrolysis (temperature and time of heat action), as well as climatic and soil chemistry conditions (Elad et al., 2011; Juriga and Šimanský, 2018).

Among the contributions to plant growth, biochar is known to potentiate the plant response to biotic stresses. Its use against airborne and soilborne fungal pathogens already showed positive effects that are attributable to interactions with soil microbes and plants rather than to the direct release of fungitoxic compounds (Bonanomi et al., 2015). Concerning the direct effects on plants, it has been suggested that biochar can induce both systemic-acquired resistant and inducing systemic resistant (Harel et al., 2012), even if the mechanisms have not been well-understood yet and the results observed so far seem to be dose dependent. In contrast, there is more robust empirical evidence that biochar can promote the development and activity of plant growth-promoting microorganisms (PGPMs), such as rhizobacteria, mycorrhizal, and other endophytic fungi. These microorganisms effectively exploit biochar porous structure to find refuge from predators, such as mites, collembolan, protozoans, and nematodes, whereas the biochar-derived organic carbon contributes to sustain their saprophytic growth (Bonanomi et al., 2018). The PGPMs in turn play a crucial role in protection against pathogens by means of competition for nutrients and space, direct parasitism, and antagonism through the production of secondary metabolites (Bonanomi et al., 2018). For example, the fungi *Trichoderma* spp. are known to be efficient competitors for space and nutrients and to rapidly colonize plant roots. Their beneficial effects on root system architecture, as well as their secondary metabolites released in the rhizosphere, promote plant growth and elicit plant responses against several pathogens (Vicente et al., 2022). Similarly, the bacteria *Bacillus* spp. can trigger plant defenses and improve plant fitness and nutrition through the production of bioactive secondary metabolites and phytohormones (Poveda and González-Andrés, 2021; Dimki et al., 2022). The presence of biochar has been shown to enhance these beneficial effects in a wide range of host plants, including horticultural crops, cereals, soybean, and woody plants, and has therefore promoted the use of this amendment as a carrier of PGPMs (Ribera et al., 2017; Ahmad et al., 2020; Sani et al., 2020; Haider et al., 2021).

The use of biochar, alone or together with PGPMs in the soil, has repeatedly proved to be effective against fungal pathogens, whereas little information is available about the possible protection from viral diseases. In this study, the effects of biochar obtained from olive pruning (certified by EUROFINS) have been evaluated on tomato plants infected by potato spindle tuber viroid (PSTVd) and tomato spotted wilt virus (TSWV), two regulated non-quarantine pests in Europe (Commission Implementing regulation EU 2019/2072; Annex IV) and included in the European and Mediterranean Plant Protection Organization (EPPO, 2022) list A2 (https://www.eppo.int/ACTIVITIES/plant_quarantine/A2_list). PSTVd, the type species of the genus *Pospiviroid* in the family Pospiviroidae (Di Serio et al., 2014), is a very damaging pathogen of a high number of Solanaceae species. Different degrees of symptom severity can be observed in tomato, ranging from leaf chlorosis and growth reduction to the loss of flower and fruit production (Owens and Verhoeven, 2017). TSWV is the type species of the genus *Orthotospovirus* (family Tospoviridae); it is responsible for severe damages to tomato cultivations, causing plant stunting and yield reduction, and blemishing the fruit with necrotic or chlorotic ringspots that make it unmarketable (Stevens et al., 1991). The impact of biochar on the virus-/viroid-infected tomato plants was assessed for a possible application in sustainable programs of disease prevention and protection. In this frame, the effects of biochar on the tomato seedling growth, as well as on the virus/viroid titer and symptom expression in infected plants, were evaluated. Besides the effects of biochar itself, the potential role as a carrier of PGPM was also investigated (using *Trichoderma* spp. and *Bacillus* spp.-based products).

## Materials and Methods

### Preliminary Evaluation of Biochar Activity

The amendment used in this study was obtained from the transformation of olive tree pruning chopped directly in the field, transformed into pellets and subsequently into biochar (by S.E.A. Company, University of Tuscia, Viterbo, Italy; Zambon et al., 2016). The physical and chemical characteristics were evaluated according to the European Biochar Certificate guidelines (EBC, 2012–2022) at the EUROFINS laboratory (Niederlassung Freiberg, Germany). The EBC analysis of the physiochemical characteristics confirmed that the biochar used in this study completely complied with the European legislation in the matter of soil improvers, thereby authorizing its use as a soil amendment (Zambon et al., 2016). All the ratios between biochar and peat reported in this study were calculated considering the dry weight of both substrates. The composition of the peat was organic component 57.4%, mineral component 42.6, pH 3.4.

#### Biochar as a Promoter of Plant Growth

Different concentrations of biochar (1, 2, 5, 10, 20, 30, 40, and 50% in ratio to peat) were evaluated in a “split-pot” bioassay in triplicate. The different concentrations were chosen to highlight the potential positive effects at low concentrations of biochar, as well as possible phytotoxic effects at high levels of biochar (>10%). Tomato seedlings were planted into small pots, each split in a first half filled up with peat only and in a second half filled up with a substrate composed of peat and biochar at different concentrations. After 30 days, the dry weight of the tomato roots was analyzed.

#### Biochar as a Promoter of Rhizobacteria in Soil

Rhizobacteria were isolated from the rhizosphere of tomato plants that were grown both in peat and the substrates containing 1, 2, 5, and 10% of biochar. Specifically, 10 g of soil was suspended in 90 ml of saline solution (0.85% NaCl), shaken for 30 min, and the soil suspensions were then 10-fold diluted and plated onto King B medium [10 g glycerol, 1.5 g K_2_HPO_4_, 1.5 g MgSO_4_.7H_2_O, 20 g Proteose peptone No. 3 (Difco), 15 g technical agar (1.5% w/v) per liter] and trypticase soy agar medium (casein peptone 15 g, soy peptone 5 g, sodium chloride 5 g, agar 15 per liter) in Petri dishes that were incubated at 28°C (Alves Silva et al., 2003).

### Biochar as a Carrier of Plant Growth Promoters (PGP)

Two commercial products of PGPMs were used in this study, namely, Remedier (Gowan, Ravenna, Italy) containing *Trichoderma gamsii* strain icc 080 and *Trichoderma asperellum* strain icc 012 (3 × 10^7^ CFU/g) and Sublic Linea Activator (Microspore, Campobasso, Italy) containing *Bacillus licheniformis* and *Bacillus subtilis* (10^9^ CFU/g). The growth of *Trichoderma* spp. was evaluated on potato starch dextrose agar (potato starch 4 g, dextrose 20 g, agar 15 g per liter) plates added with different concentrations of biochar (1, 2, 5, and 10%) with diameter < 1 mm. Specifically, five different isolates of the two *Trichoderma* species (icc 080 and icc 012 obtained from Remedier product) were plated, and the mycelial growth was evaluated at 24, 48, and 72 h. The growth of *Bacillus* spp. was evaluated using both *B. subtilis* and *B. licheniformis*. Specifically, colonies of *B. subtilis* (2.3 × 10^5^ CFU/ml) and *B. licheniformis* (7 × 10^5^ CFU/ ml) were grown (120 rpm, 26°C ± 1°C) in liquid broth added with biochar (1, 2, 5, and 10%) with <1 mm of diameter, and then reisolated and counted after 48 h. The survival of *Bacillus* spp. and *Trichoderma* spp. on biochar was also estimated after 90 days from the preparation. Biochar was stirred for 2 h in aqueous suspension with either “Sublic” or “Remedier,” the substrate was then dried and kept at room temperature. Reisolations were performed at 10, 20, 60, and 90 days after the treatment.

### Experimental Design

In a two-year research, the effects of biochar and PGPMs at different concentrations were evaluated on PSTVd- and TSWV-infected tomato seedlings in three independent experiments per pathogen.

Tomato seedlings (cultivar Roma) were grown in peat for 30–40 days following an organic cultivation. Then, the seedlings were transplanted in six substrates, including the control (A0) containing only peat (Table 1). Substrate A was prepared by combining peat and biochar at 1, 2, 5, 10, and 15%. Substrates B1 and B2 were the same as substrate A with the addition of *Bacillus* spp. and *Trichoderma* spp., respectively, at different concentrations that depended on the adsorption of those microorganisms on the biochar. Specifically, the initial concentration of *Trichoderma* spp. was 2 × 10^4^ CFU/g and of *Bacillus* spp. of 3.5 × 10^6^ CFU/g. In substrates C1 and C2, *Bacillus* spp. and *Trichoderma* spp. were added to the peat without biochar at the same concentration of the B1 and B2 media, respectively.

**Table 1:** Composition of the six basic substrates used in the experiments.

	**Peat**	**Biochar**	***Bacillus* spp**.	***Trichoderma* spp**.
A0	+	–	–	–
A	+	+	–	–
B1	+	+	+	–
B2	+	+	–	+
C1	+	–	+	–
C2	+	–	–	+

*Substrates A, B1, and B2 were prepared with five different biochar concentrations (1, 2, 5, 10, and 15%)*.

Mechanical inoculations of the virus/viroid were performed on transplanted seedlings at four/five true leaf stage. An isolate of PSTVd (isolate Sj1—GenBank Accession No. HQ452413, belonging to the pathogen collection of the CREA-DC) and an isolate of TSWV (VE-TSWV-not breaking resistance-RB), provided by the Institute for Sustainable Plant Protection—National Research Council, Italy, were chosen as the source of inoculum. Sap for inoculum was prepared by grinding infected material in sterilized phosphate buffer (0.1 M, pH 7.2) at the concentration of 1:20 W/V. In addition, 40 μl of the solution was used to inoculate the two last expanded apical leaves (20 μl each) previously covered by abrasive powder (celite). The infectious status of all the inoculated plants was tested at 30 days post inoculation (dpi) when the first symptoms appeared.

All experimental trials were conducted in a greenhouse at 20–24°C with a 12–14-h photoperiod.

### Evaluation of Biochar Activity on Systemic Pathogens

In the first experiment (trial 1), plants were grown onto all the typologies of substrates (A, B1, B2, C1, and C2) supplied with biochar at 1, 2, and 5%. Each treatment was carried out on 3 and 6 seedlings inoculated with PSTVd and TSWV, respectively. The second experiment (trial 2) followed the same scheme as trial 1, but nine PSTVd and nine TSWV inoculated seedlings were used per treatment and the biochar concentration was increased up to 10 and 15%. In both trials 1 and 2, the PSTVd and TSWV titer was measured at 30^th^ dpi. A third experiment (trial 3) was carried out using only the combination of biochar/PGPMs concentrations selected for the best effects observed against the viroid/virus in the previous experiments. In this trial, the seedlings were allowed to grow to an adult stage, and 12 inoculated plants per treatment/per pathogen were evaluated only for growth and symptoms expression at 90^th^ dpi.

For each experiment and substrate typology, three healthy (non-inoculated) plants were used as control in order to distinguish the viroid/virus symptoms from possible alterations due to the different combinations/concentrations of biochar or other environmental factors.

#### Symptom Visual Examination

Starting from 1 week after the inoculation, the plants of the first and second experiments were periodically inspected by visual examination for systemic symptoms development and plant growth rate until 30^th^ dpi when leaf samples were collected for virus/viroid titer quantification. In the third experiment, the plants were maintained until 90^th^ dpi for the symptom examination only. Specifically, three classes of severity were defined for the symptoms induced by each pathogen at 90 dpi. For PSTVd, the classes were no symptoms; mild symptoms corresponding to leaf mosaic and curling; and severe symptoms corresponding to necrosis of leaves, shortened internodes, apical bunching, and stunted growth. For TSWV, the classes were no symptoms; mild/middle symptoms corresponding to chlorotic and/or necrotic spots on leaves with normal growth of the plants; and severe symptoms corresponding to necrotic spots, leaf distortion, apical necrosis, and stunted growth of the plants.

#### Pathogen Detection and Relative Quantification

Three leaf samples corresponding to the basal, medium, and apical positions were collected at the 30^th^ dpi from each plant in trials 1 and 2. Total RNA (TRNA) was extracted from 0.1 g of powdered leaves (obtained using liquid nitrogen) using Spectrum Plant Total RNA kit (Sigma, Deisenhofer, Germany) and TRNA tissues and cells kit (Danagen, Spain) for PSTVd and TSWV, respectively.

The detection of PSTVd was performed by the end-point RT-PCR according to Faggioli et al., (2013), and the quantification was performed by RT-qPCR according to Boonham et al. (2004). The detection of TSWV was achieved using a DAS-ELISA Kit (Loewe Biochemica GmbH, Germany), and the quantification was performed by RT-qPCR using the primers and probes reported in Table 2. The relative quantification of both pathogens was carried out using the cytochrome oxidase gene (Weller et al., 2000) as endogenous control.

**Table 2:** Sequence of primers and probe used for TSWV quantification.

**Name**	**Sequence (5′-3′)**	**Position**
TSWV333F	AAGCTACCTCCCAGCATTATGG	2,352–2,373
TSWV415R	TCTCACCCTTTGATTCAAGCCTAT	2,411–2,434
TSWV356T	6-FAM-AAGCCTCACAGACTTTGCATCATCAAGAGG-BHQ1	2,375–2,404

*Position is referred from the sequence LC549181 of the NCBI database*.

All RT-qPCR tests were performed including 2 μl of total RNA to the following 18 μl reaction mixture, namely, 1X TaqMan® RT-PCR Mix and 1X TaqMan® RT Enzyme Mix (TaqMan® RNA-to-CT™ 1 Step Kit from Thermo Fisher Scientific, MA, USA), 0.8 μM primer forward; 0.8 μM primer reverse; 0.2 or 0.4 μM, for PSTVd and TSWV, respectively, of the probe labeled in FAM/BH1 (BioFab Research, Italy). The same mixture was used for the endogenous control amplification; also, this probe was labeled FAM/BH1 (BioFab Research, Italy). All amplifications were performed in an Abi7500Fast thermocycler (Life Technologies, CA, USA) using the cycling conditions 10 min at 50°C for reverse transcription followed by 5 min at 95°C; amplification was performed for 40 cycles with denaturation for 10 s at 95°C and annealing combined with extension for 30 s at 60°C.

##### Data Analysis

The comparison of the concentrations of the pathogens obtained from the plants grown on different media was done using the −ΔΔCt method, subtracting the cycle thresholds (Cts) of treated samples (A, B1, B2, C1, and C2) with non-treated infected plants (A0). Before applying this method, primers and probes were tested to demonstrate that the amplification efficiency of the diagnostic methods was approximately the same as the endogenous control (Livak and Schmittgen, 2001) and the absolute value of the slope of each test was determined to be <0.1 (Supplementary Material).

Results from the calculation were analyzed by ANOVA (Montgomery, 2012). Mean squares of main effects and interactions were compared by *F*-tests (95% of significance) with error variance estimated by three replicates of test condition. ANOVA was performed for each monitored variable (concentration of biochar, presence/absence of microorganisms). Significant results were analyzed using a *post-hoc* test (*t*-test).

## Results

### Preliminary Evaluation of Biochar Activity

**Figure 1:**
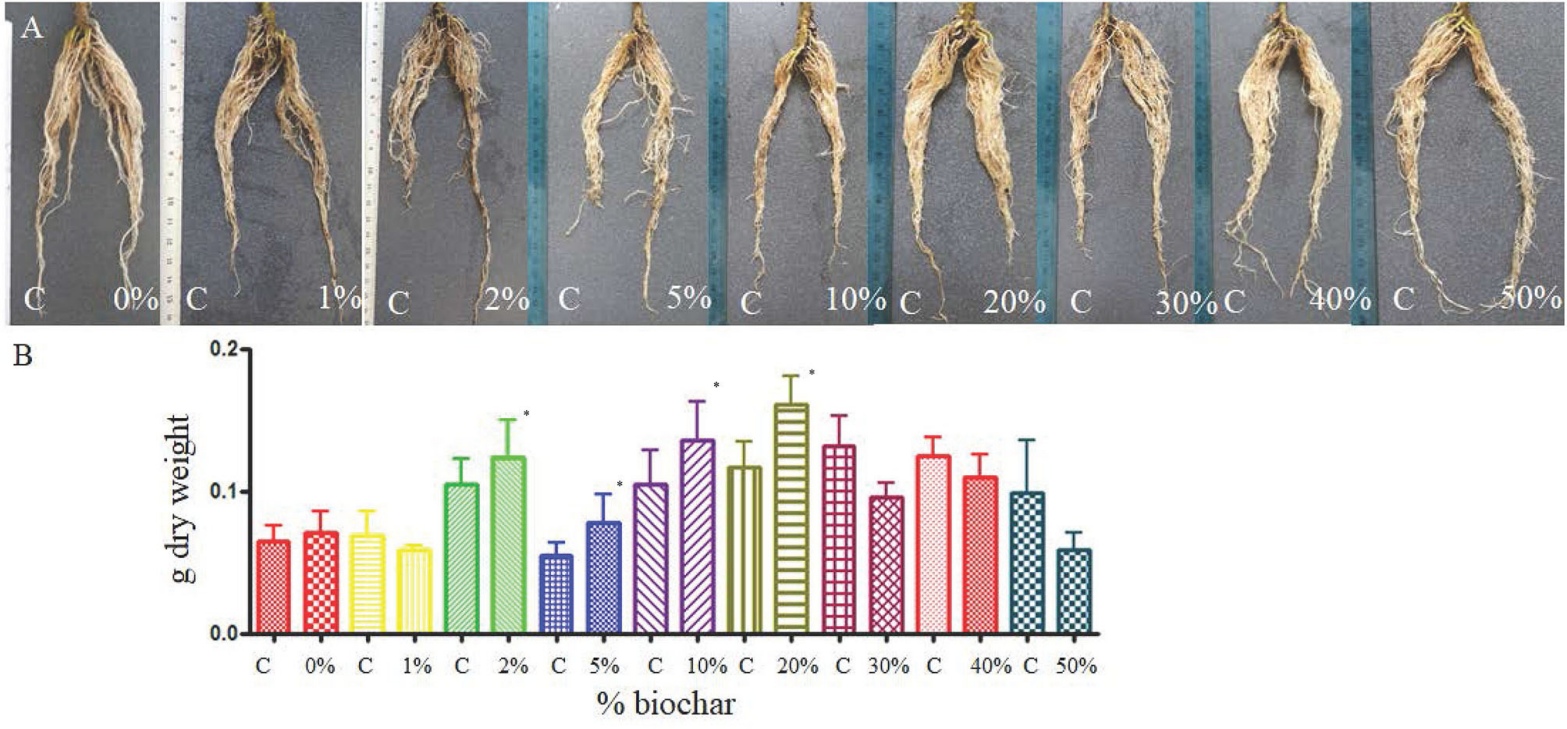
Root of tomato seedlings **(A)** grown half in peat (C, control) and half on a substrate containing different concentrations of biochar (from 0 to 50%) in a split pot test. **(B)** Dry weight of the same roots grown half in peat (C) and half in biochar at different concentrations. *p < 0.05.

The split-pot test showed that biochar can positively affect the development of tomato roots 30 days after planting, and the dry weight of the roots grown in biochar at the concentration from 2% to 20% was significantly higher than the control (roots grown in peat only) (Figures 1A,B).

**Figure 2:**
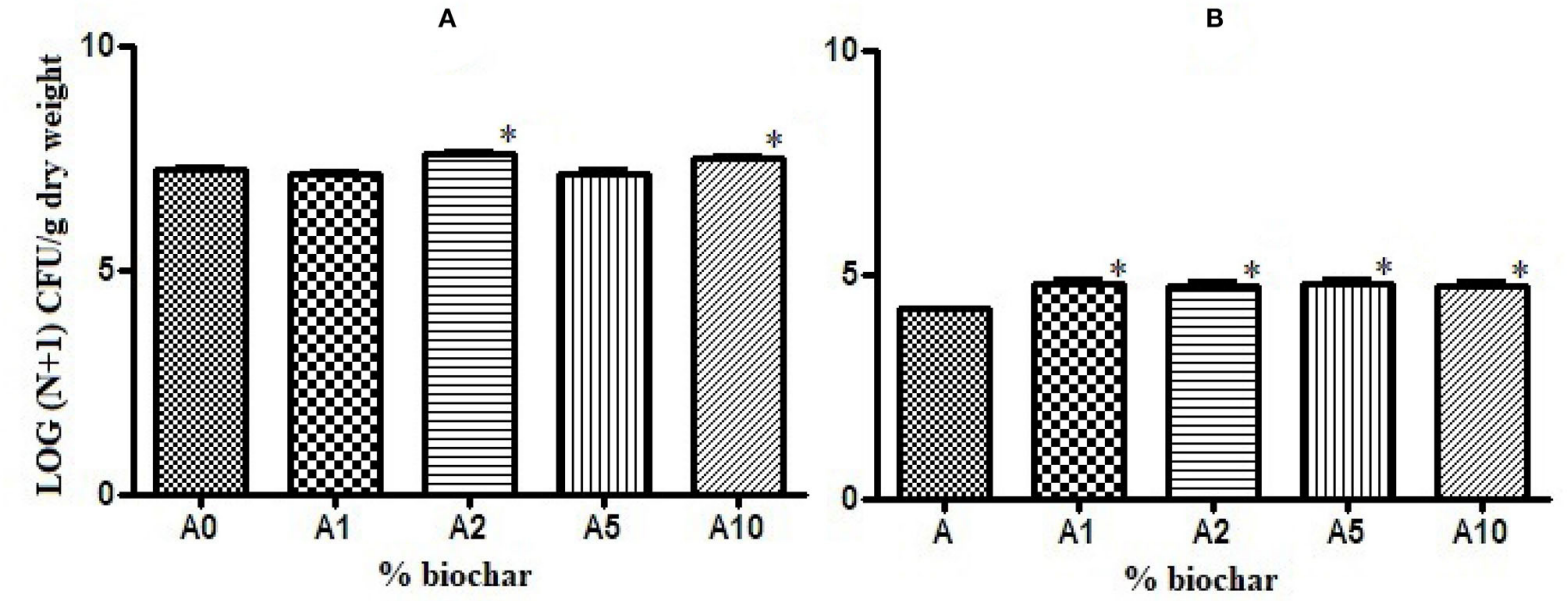
Effect of different concentrations of biochar on the total bacterial load **(A)** and the sporogenic bacteria concentration **(B)** of the rhizobacteria isolated from the tomato roots. *p < 0.05.

Biochar showed a positive effect on the growth of rhizobacteria present in the rhizosphere of the tomato seedlings. The total bacterial load was significantly higher in substrates with 2 and 10% of biochar (A2 and A10) than in the control substrate (A0), (Figure 2A), and the charge of sporogenic bacteria significantly increased in all the biochar substrates (Figure 2B).

Both *Trichoderma* spp. and *Bacillus* spp. were successfully grown in the proper media added with 1, 2, 5, and 10% of biochar, and they were reisolated after keeping the samples for 90 days at room temperature, suggesting that the amendment has no antimicrobial effects (data not shown).

### Effect of Biochar on the Response of Tomato Systemically Infected Plants

The effect of the biochar on the plant systemic pathogens was ascertained by both symptom evaluation and virus/viroid quantification with respect to the expression of a stable endogenous control. Primers and probes were tested before to ascertain the comparability of the efficiency of the quantification protocols (see Supplementary Material).

#### Potato Spindle Tuber Viroid

**Figure 3:**
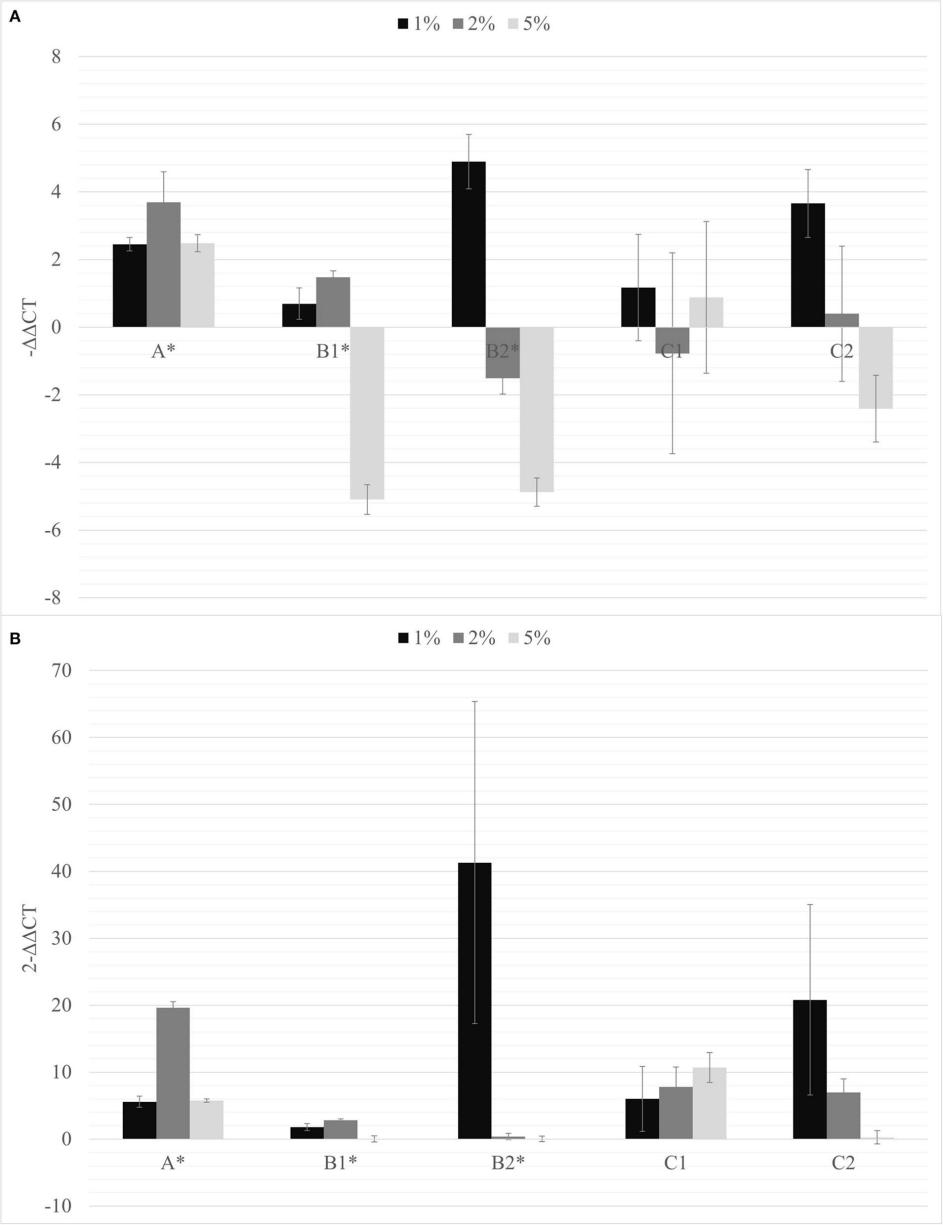
The –ΔΔCt **(A)** and fold change in PSTVd replication (2^−Δ*ΔCt*^) **(B)** mean values obtained for all the biochar percentages (1% black; 2% dark gray; 5% light gray) are reported. *p < 0.05.

Considering the effects of biochar observed on the growth of tomato root and on the growth of rhizobacteria and PGPMs, biochar at 1, 2, and 5% was first added to the A, B1, and B2 substrates and the same PGPM concentration used in B1 and B2 was added to peat into C1 and C2 substrates. All the PSTVd-inoculated tomato plants growing onto these different substrates resulted to be symptomatic and positive to the viroid after 30 dpi (data not shown) and were subjected to the viroid titer quantification (Figure 3).

**Figure 4:**
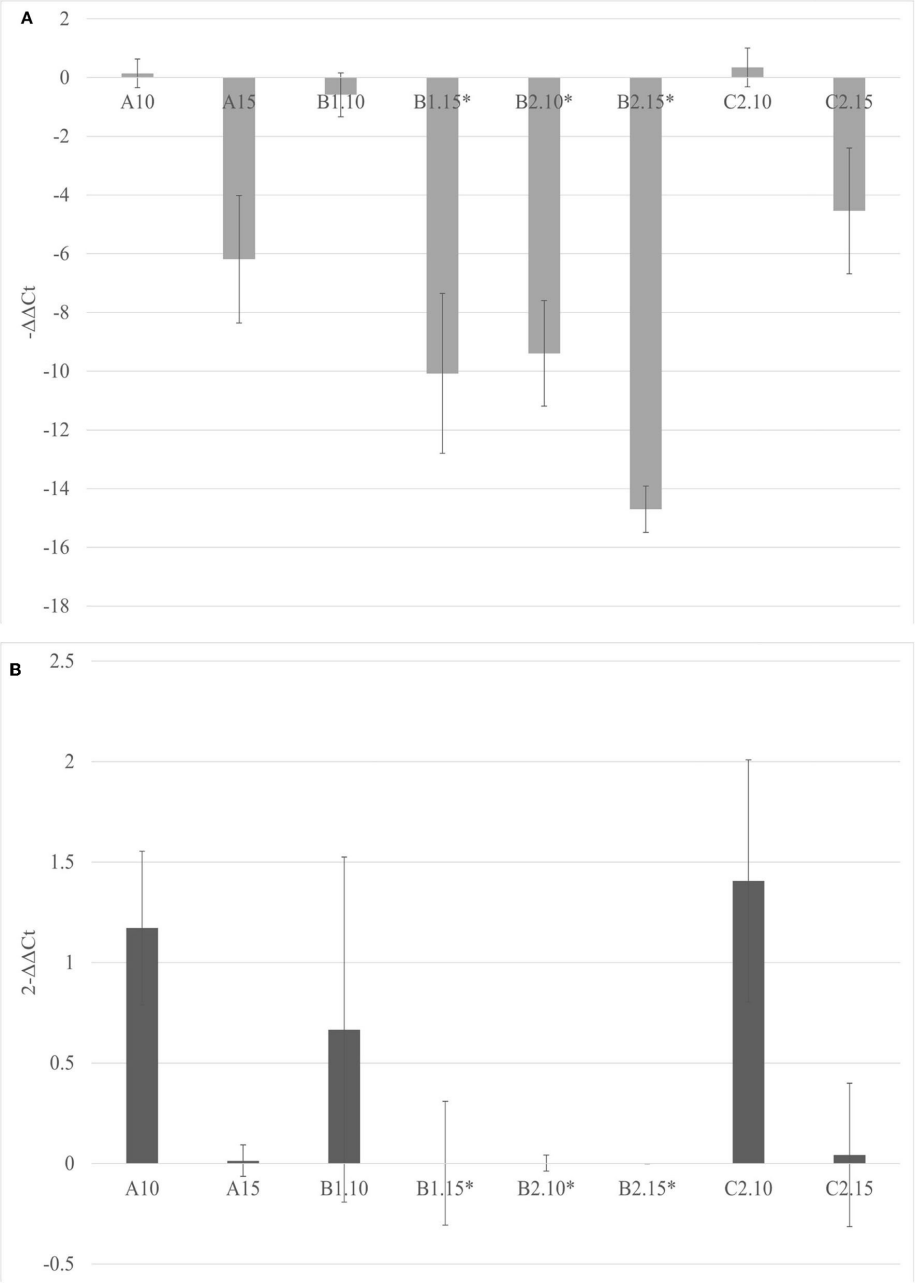
The –ΔΔCt **(A)** and fold change in PSTVd replication (2^−Δ*ΔCt*^) **(B)** mean values obtained for all the theses are reported in the left and right, respectively. *p < 0.05.

The soils containing only *Bacillus* spp. (C1) and *Trichoderma* spp. (C2) were not able to inhibit the viroid's replication, and the soil containing only biochar (A) even promoted the replication of the viroid compared to the control. On the contrary, the soils with 5% of biochar in combination with the PGPMs (B1 and B2) significantly reduced the replication of PSTVd compared to the control (A with 5% biochar). According to this, soils with higher concentrations of biochar, 10 and 15%, were prepared and new tests were made inoculating 9 new healthy tomato plants for each substrate. Also in these trials, all the plants tested positive for PSTVd after 30 dpi (data not shown) and were analyzed for titer quantification. In all the theses, the viroid titer decreased compared to the control, but this reduction was significant only in plants grown in the substrate containing 10% of biochar together with *Trichoderma* spp. (B2.10), as well as in the substrate containing 15% of biochar added with *Bacillus* spp. and *Trichoderma* spp., respectively (B1.15 and B2.15) (Figure 4).

**Figure 5:**
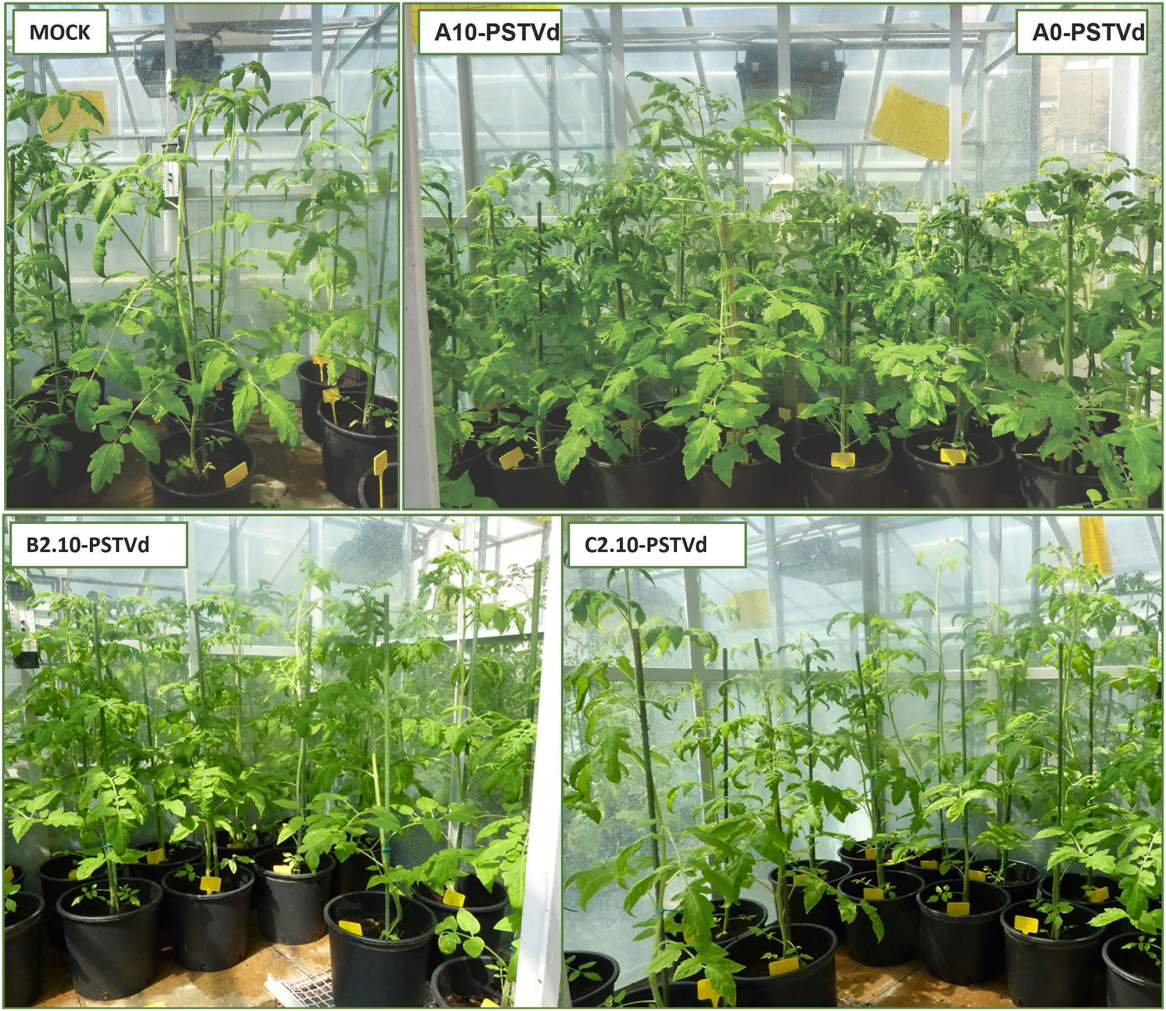
PSTVd symptoms observed on tomato plants growth on different substrates: MOCK-controls inoculated with buffer only; A10-PSTVd, A0-PSTVd showing severe symptoms: shortening of internodes, bunched leaves and shoots on the top of the plants, and stunted growth; B2.10 and C2.10-PSTVd showing mild symptoms: curling leaves and distortion.

**Figure 6:**
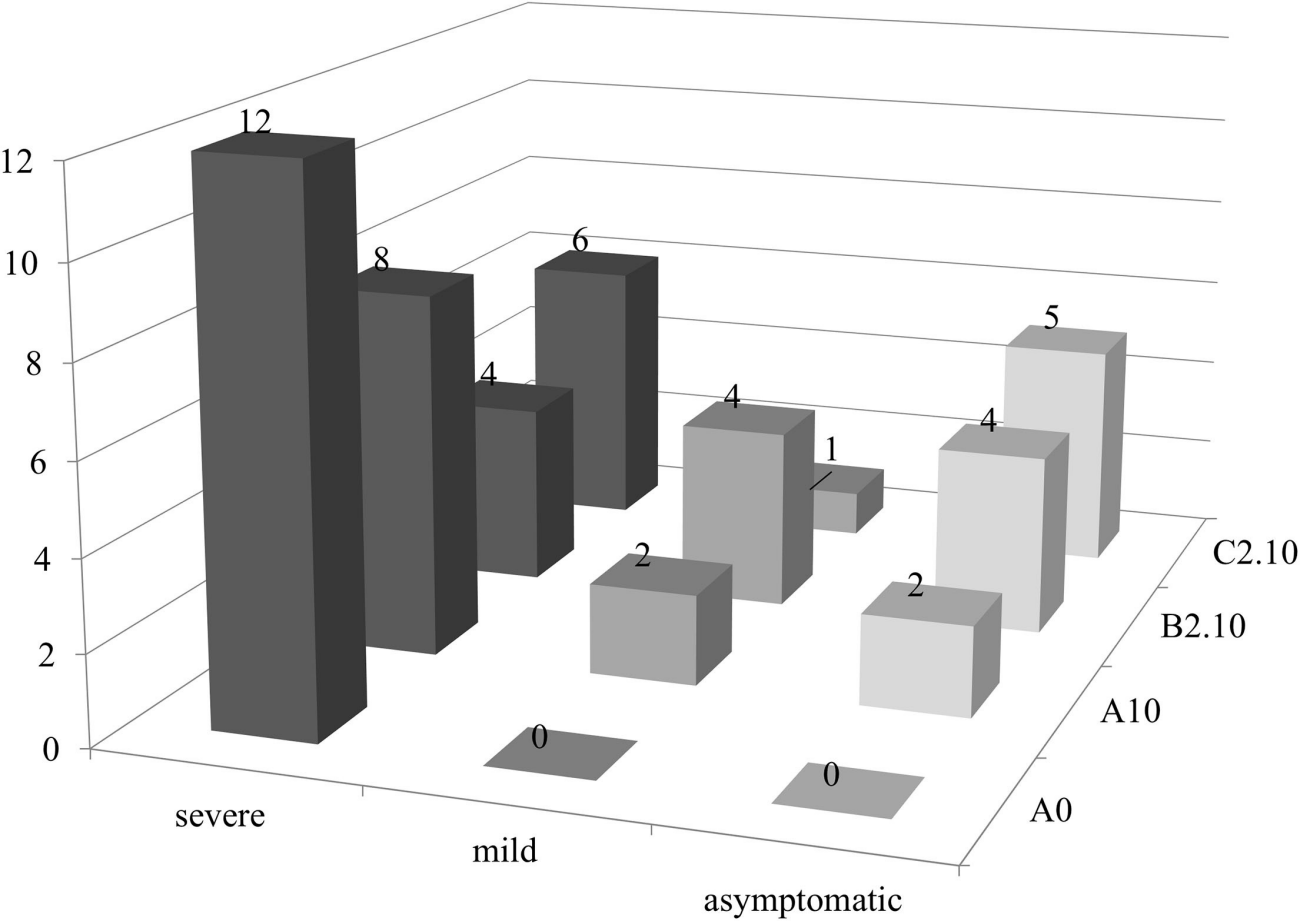
PSTVd symptoms modulation (no. of plants evaluated according to the expression of symptoms for each substrate in this study). Black, severe symptoms; dark gray, mild symptoms; light gray, no symptomatic plants.

A possible effect of biochar and PGPMs on the expression of PSTVd symptoms was inspected growing 12 tomato plants for each substrate for 90 days. The plants were grown on substrate B2.10, which resulted in the best combination to reduce the titer of the viroid, and on substrates A0, A10, and C2.10 as controls. All the inoculated plants tested positive for the viroid, but the symptom modulation varied according to the different substrates (Figures 5, 6). All the plants grown in substrate A0 (only peat) showed severe symptoms (Figure 5 panel A0-PSTVd) consisting of shortened internodes, bunched lives and shots in the top of the plants, and stunted growth. Similar symptoms were also obtained from the plants transplanted on A.10 substrate (peat and biochar) (Figure 5—panel A.10 PSTVd). Plants growing in C2 substrate showed a variability of symptoms, from mild to severe symptoms. Plants grown in the soil containing biochar and *Trichoderma* spp. (B2.10) had the best response to the viroid infection showing mild symptoms, mainly curling and distortion of the leaves, or no symptoms (Figure 5 panel B2.10). The distribution of the 12 plants per thesis among the three classes of symptoms severity is reported in Figure 6.

#### Tomato Spotted Wilt Virus

In the first experiment carried out with the lowest concentrations of biochar (1, 2, and 5%), both symptom observation and virus detection at 30 dpi showed that not all the inoculated plants were infected. The distribution of infection was not uniform among the 6 seedlings of each treatment (Table 3) and did not allow a direct comparison to the control A0 (3 infected plants out of 6 inoculated plants). Nevertheless, some differences among substrates are evident (Table 3), such as the absence of infected plants in the B2 (biochar plus *Trichoderma* spp.) soil containing 2 and 5% of biochar, and in the C1 soil containing *Bacillus* spp. at the same concentration used in addition to 1 and 2% of biochar in B1.

**Table 3:** Number of TSWV-infected plants out of the six inoculated plants grown onto the different substrates.

**A0**	**Biochar %**	**A**	**B1**	**B2**	**C1**	**C2**
3	1	4	2	3	0	4
	2	4	3	0	0	2
	5	4	2	0	5	4

**Figure 7:**
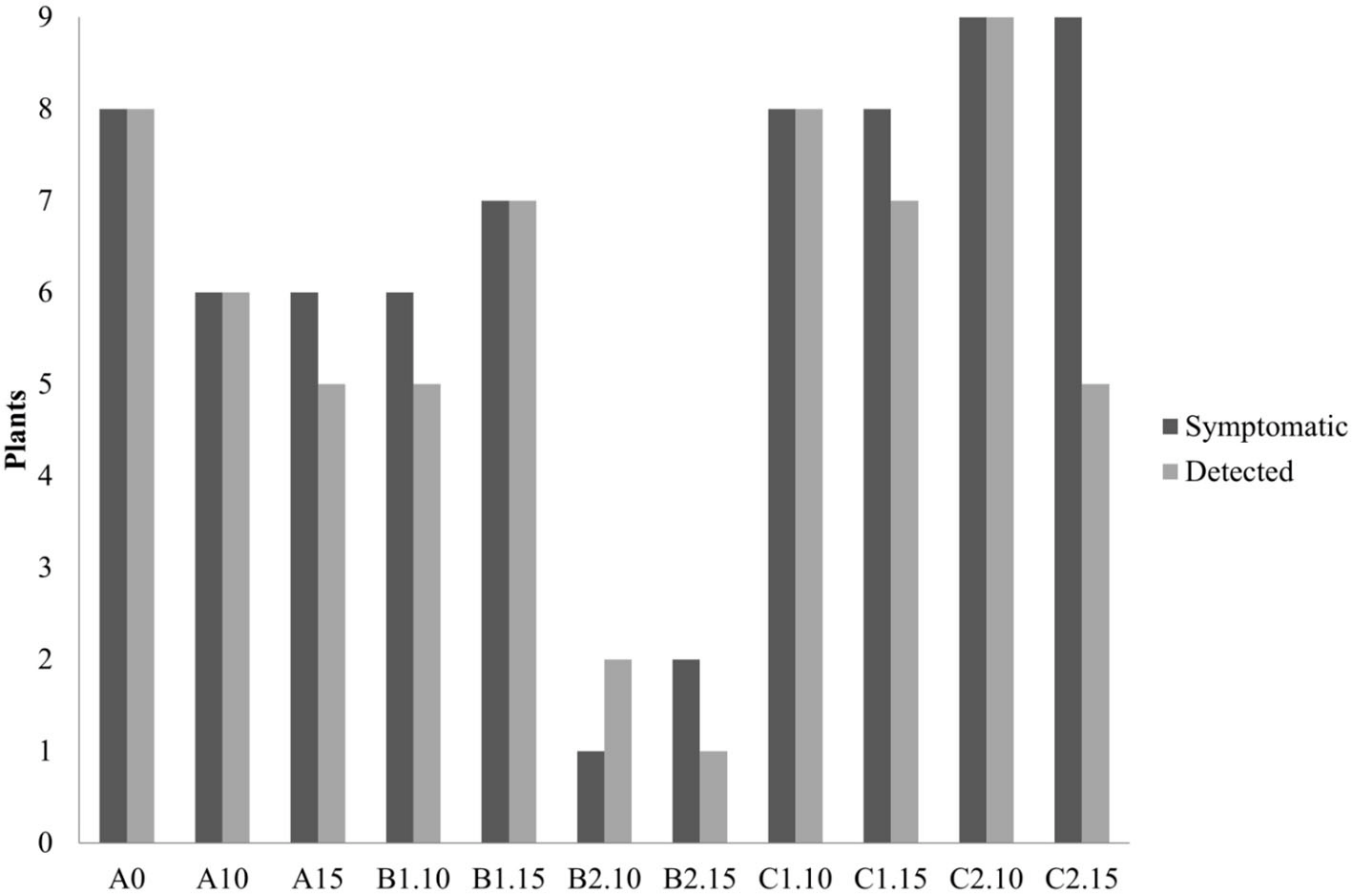
Number of symptomatic and TSWV-positive tomato seedlings grown on different substrates (A0 control-only peat; A, peat combined with biochar; B1, peat combined with biochar and *Bacillus* spp.; B2, peat combined with biochar *Trichoderma* spp.; C1, C2, peat combined only with *Bacillus* spp. or *Trichoderma* spp., respectively), numbers 10 and 15 represent the concentrations. *p-Value <0.05.

**Figure 8:**
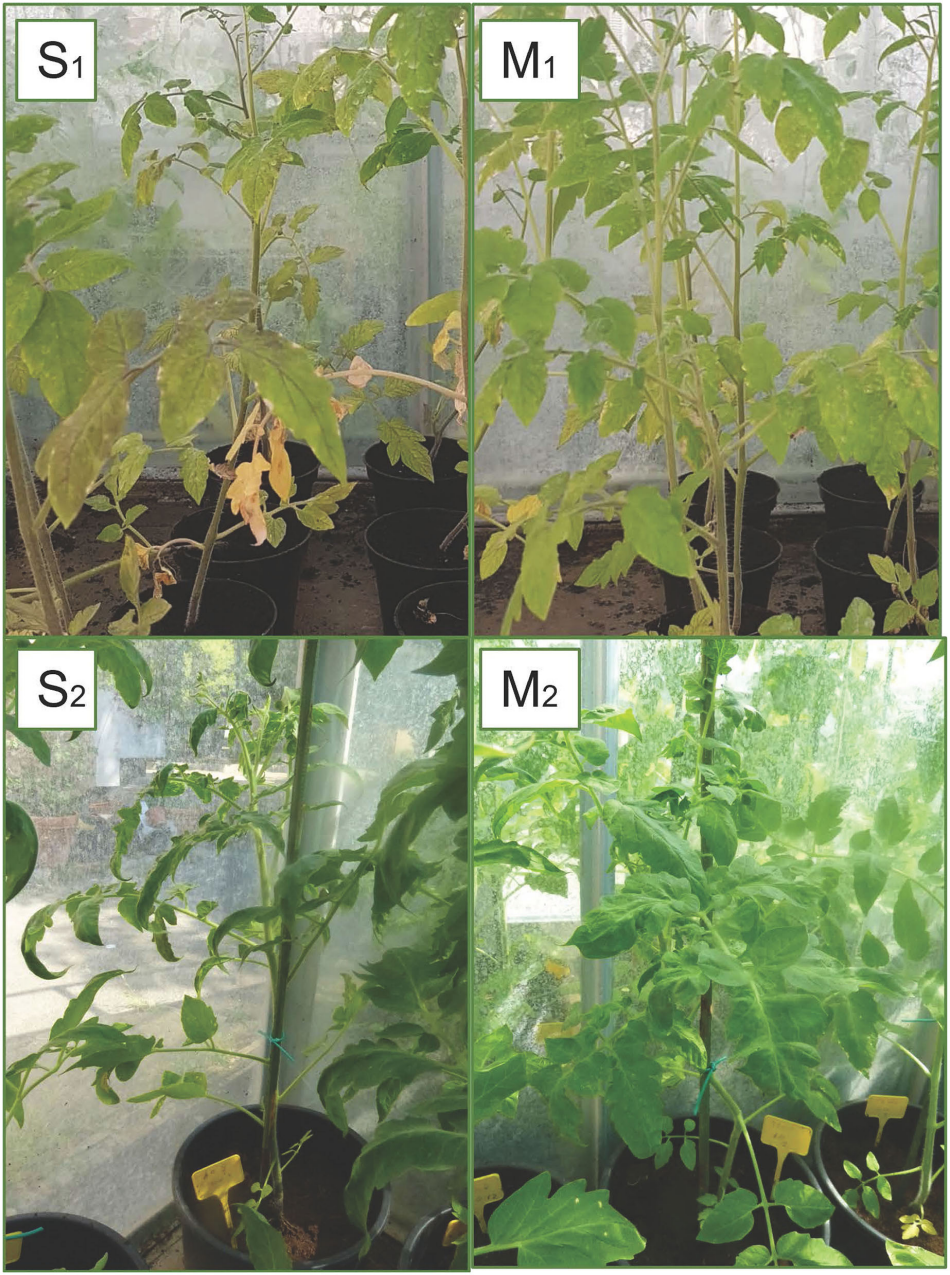
Symptoms of TSWV observed in plants grown on different substrates: severe symptoms with typical necrotic spots and bronzing of leaves **(S1)** and reduction in the plants growth **(S2)** in A0 (only peat); mild symptoms with chlorotic spots or chlorotic leaves **(M1)** and middle symptoms **(M2)** observed in substrates with biochar (A10 and B2.10) (with peat, biochar, and *Trichoderma* spp.).

**Figure 9:**
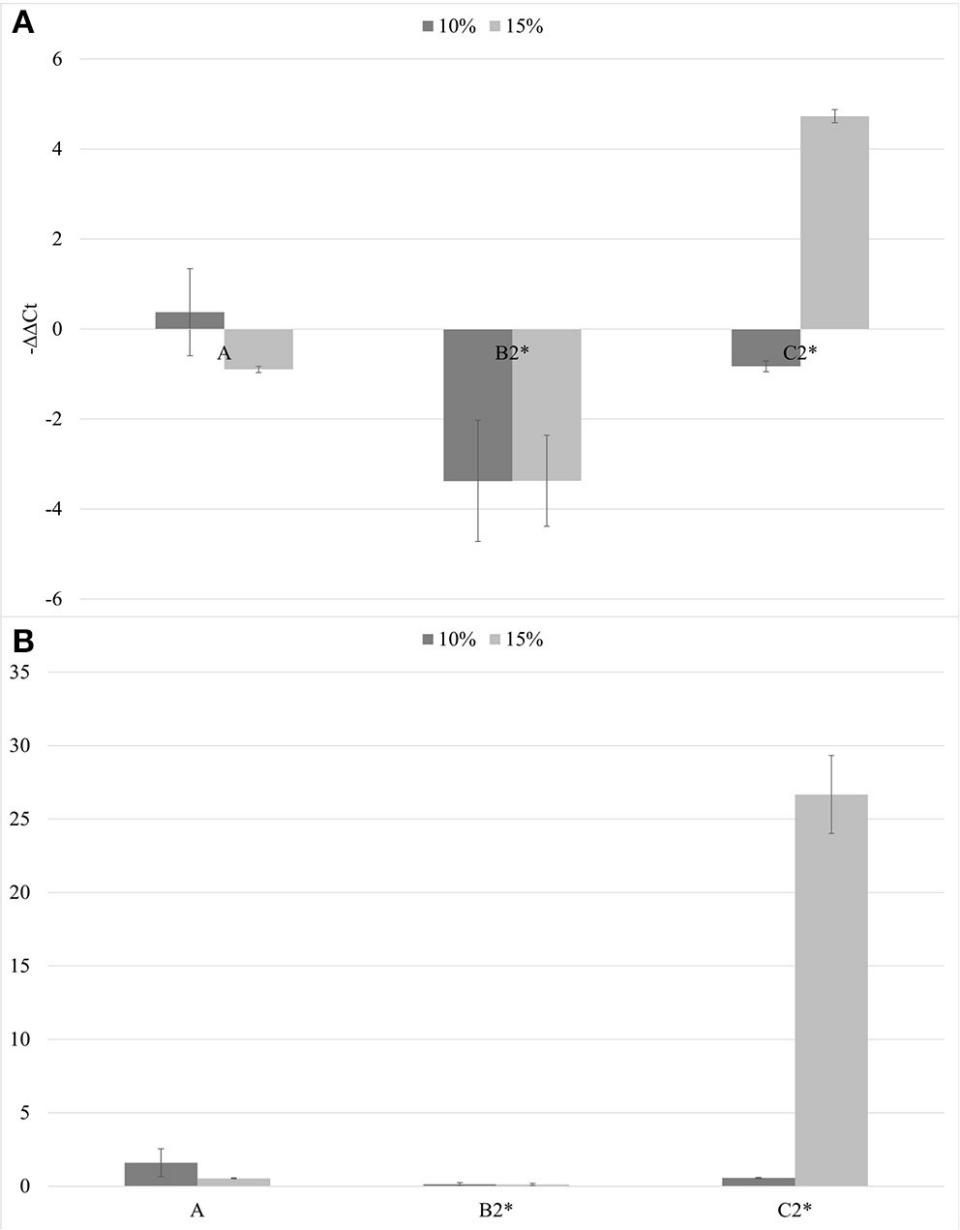
The –ΔΔCt results **(A)** and fold change in TSWV replication (2^−Δ*ΔCt*^) **(B)** mean values obtained for all the theses (10% in gray and 15% in light gray). *p < 0.05.

In the second trial, again all the substrates were evaluated, increasing the number of seedlings (9 per each treatment) and using higher concentrations of biochar, 10 and 15%. ELISA test at 30^th^ dpi showed a different number of infected plants grown on the different substrates (Figure 7). The highest number of the infected plants compared to the inoculated ones (8/9) was obtained in the control substrate (A0-only peat) and in the substrates containing PGPMs only (C1 and C2). In particular, in the C1 substrate (peat and *Bacillus* spp.), the infected plants were 8/9 and 6/9, and in the C2 substrate (peat and *Trichoderma* spp.) the infected plants were 6/9 and 8/9. The number of infected plants decreased (5/9) in substrate A containing biochar at both 10 and 15%, and in substrate B1 (peat, biochar, and *Bacillus* spp.) at 10% concentration. Finally, B2 substrate containing both 10 and 15% of biochar and *Trichoderma* spp. showed a reduction in the number of positive samples that was significant compared to the other substrates (1/9 and 2/9, respectively). Symptom expression at 30^th^ dpi also varied according to the different substrates. The most severe symptoms, consisting of the typical necrotic spots and bronzing of leaves, were observed in treatment A0 (mock control) in all infected plants (Figure 8-S1). In the other substrates, the symptoms consisted generally of chlorotic spots or chlorotic leaves (Figure 8-M1) without difference in symptoms severity but in the rate of number of symptomatic/infected plants (Figure 7). As the number of infected plants obtained in this experiment was higher than in the first one, the real-time RT-PCR analyses for the relative quantification of the viral titer in plants grown on different substrates were possible. The TSWV titer was measured for plants grown in substrate B2 (peat, *Trichoderma* spp., and biochar) at both concentrations 10 and 15% that showed the minor number of infected plants and for the plants grown on the respective substrate controls A10 (peat and biochar) and C2 (peat and *Trichoderma* spp.). The viral titer was lower in plants grown in substrate B2.10 (biochar and *Trichoderma* spp.) compared to plants grown in substrates A10 (peat and biochar) and C2.10 (peat and *Trichoderma* spp.); the titer decreases to about 40,000 times in B2.10 with respect to the infected plants of the control A0 (Figure 9).

The third experiment, for symptom evaluation at 90^th^ dpi, was carried out using the A and B2 soil with a biochar concentration of 10%, and A0 and C2 soils served as controls. Again, only a few inoculated plants resulted to be positive to TSWV: 1/10, 3/12, 5/12, and 3/12 for the substrate A0, A10, B2.10, and C2.10, respectively. Severe symptoms, such as stunting of plants, leaf malformation, and necrosis, were observed in the infected plants grown on A0 and C2 substrates, whereas some infected plants grown on biochar substrates were not severely affected by infection and showed good foliage conditions (1/3 and 2/5 plants of A.10 and B2.10, respectively) (Figure 8-S2 and M2).

## Discussion

The use of biochar as soil amendment can potentially influence the interactions between plant and beneficial microbes, and has a positive impact on the response to pathogen diseases. On the one hand, the enrichment of aromatic molecules originating during biomass pyrolysis makes biochar an organic material capable of stimulating plant growth. On the other hand, the porous structure can physically sustain soil microbe colonies in sites that are not reachable by grazers or predators, making biochar a potential carrier for a variety of beneficial microorganisms (Bonanomi et al., 2018).

Positive effects on both plants and soil microbes were observed also with the biochar used in this study. This product was obtained from olive pruning and has all the characteristics to be used as soil amendment, namely, basic pH and low presence of heavy metals and polycyclic aromatic hydrocarbons. The experiments carried out on tomato seedlings confirmed that this biochar is suitable to promote the root growth to the concentration of 20%. Moreover, no antimicrobial effects were observed at the tomato rhizosphere level on the beneficial soil bacteria, whose growth seemed to be even improved. Similarly, biochar did not show any negative effect on PGPMs either on bacteria (*Bacillus* spp.) or fungi (*Trichoderma* spp.), and it promotes the survival of these microorganisms for a long time after the colonization.

The potential role of biochar in enhancing the plant response was tested on tomato plants infected by systemic pathogen agents. The disease suppression activity is already known for soilborne pathogens, mainly fungi, whereas a few studies have been carried out on plants infected by airborne pathogens, and very limited information is available on virus- and viroid-induced diseases (Bonanomi et al., 2020). The results obtained on PSTVd-infected tomatoes showed that the biochar effect is dose-dependent and depends on the presence of PGPMs. The addition of the sole biochar into the soil did not significantly affect the viroid titer and symptoms expression, and even contributed to increase the PSTVd titer at the lowest concentrations. This confirms that, with equal biomass composition and soil chemistry, certain concentrations of biochar can induce a U-shaped dose/response curve and accelerate the plant disease, as already observed for other pathosystems (Frenkel et al., 2017). When proper concentrations of PGPMs were added to biochar, a disease suppression activity was observed. Notably, the addition of *Bacillus* spp. to 15% biochar, as well as the addition of *Trichoderma* spp. to both 10 and 15% biochar, induced a significant reduction in the PSTVd titer in tomato. The presence of *Trichoderma* spp. in the biochar-added soil also contributed to the plant response to infection, inducing only mild or no PSTVd symptoms. Therefore, 10–15% biochar in combination with *Trichoderma* spp. potentially showed a positive activity addressed to the tomato-PSTVd pathosystem since this treatment can sensibly reduce the replication of PSTVd and the expression of symptoms even if it is not able to stop the start of infection.

The data obtained for TSWV were not linear, and the first and third experiments did not provide a significant number of infected plants in the mock treatment without biochar (A0), so the results obtained by comparing the other different substrate combinations could not be considered valid. This extreme variability is probably due to the lability of TSWV that, even if a fresh inoculum was prepared for each thesis within the same experiment, could have a different titer and infection capacity in different theses/trials. Despite only the second experiment being considered valid, according to the controls, the general aspect of the plants treated with biochar yield better results than the others, with an increasing growth rate, even when infected with TSWV. Therefore, biochar could have a role in preventing also the TSWV infection as it did not occur in PSTVd. The experiments suggest that the use of biochar, particularly in combination with *Trichoderma* spp., could induce plants to have a defense response to the virus by blocking or recovering the infection in the first step after inoculation. These positive effects against TSWV confirmed the results obtained by Bonanomi et al. (2020), who tested different organic amendments and showed that biochar combined with alfalfa or manure was among the most effective soil treatments to control the disease caused by this virus in tomato plants.

The beneficial effects of biochar and *Trichoderma* observed on PSTVd- and TSWV-infected tomato plants are consistent with previous studies performed in several stress conditions. It was observed that biochar favors the hyphal growth and elongation of *Trichoderma*, which in turn improves the root system architecture of the tomato plants and contributes to the increase in the foliar area and secondary roots (Chacón et al., 2007). This results in a better uptake of mineral nutrients and photosynthesis efficiency, as demonstrated by the increase in antioxidant contents and minerals in tomato shoots and fruits as well as by the upregulation of various genes associated with photosynthesis observed after biochar-*Trichoderma* applications on tomato. These beneficial effects on tomato growth and nutrition are more evident when *Trichoderma* and biochar are supplied in combination in the soil rather than either alone. The synergistic action finally results in higher crop yield and fruit quality, as well as in a boosted defense response to several abiotic stresses, parasites, and pathogens, such as *Fusarium oxysporum* (Hasan et al., 2020; Sani et al., 2020 Arshad et al., 2021). Such a virtuous cycle was likely launched also in the PSTVd-infected tomato plants treated with 10–15% biochar and “Remedier,” leading to a reduction of both symptom severity and viroid titer.

Similar beneficial effects on tomato were reported also when *Bacillus* spp. were added to biochar (Arshad et al., 2021; Rasool et al., 2021a). For example, the combination of biochar and *B. subtilis* was showed to stimulate not only the plant growth but also the response to the soil-inhabiting fungus *Alternaria solani*, as revealed by the overexpression of defense-associated genes, such as salicylic acid-related PR genes, and the significant reduction of the early blight symptoms (Rasool et al., 2021a,b). Such effect was not so evident in this study since only the addition of *Bacillus* spp. to 15% biochar was effective in reducing the titer but not the symptoms of PSTVd. This may be due to the composition of the soil that included the peat as the main substrate. The presence of peat could have contributed to acidify the pH of the soil and thus create a medium that is not optimal for the growth of *Bacillus* spp. (Gauvry et al., 2021).

This study provides evidence that biochar can promote the root growth and the general fitness of tomato plants and contribute to prime the defense response against systemic pathogens, such as PSTVd and TSWV. The most significant effects on both virus/viroid symptoms and titer were observed when biochar was combined with PGPMs, especially *Trichoderma* spp. This confirms that biochar is an efficient carrier of beneficial microorganisms by playing a protective role and providing nutrients for microbe growth. These promising results have to be confirmed with further experiments, particularly using a more stable and easily transmissible virus as the emerging tomato brown rugose virus (Salem et al., 2016; Luria et al., 2017). The pathogenetic mechanisms that regulate the responses of plants to systemic pathogens in the presence of biochar need to be further elucidated as well. Anyway, the overall results encourage us to develop novel biocontrol products based on the synergistic combination of biochar and PGPMs. A regular supplement of biochar and *Trichoderma* in the soil can likely contribute to the sustainable management of plant viral diseases for which no curative methods are available. As the biochar activity observed in this study resulted to be dose-dependent, different types of biochar with different biomass composition should be tested to identify the most suitable concentration for each crop-pathogen system. The use of biochar in sustainable agriculture is also strongly encouraged because of its involvement in carbon seizing and greenhouse gasses reduction to the point that it has been included in the CO_2_-reducing techniques suggested in the G20 meeting of agricultural chief scientists in 2019 (Meeting of Agricultural Chief Scientists (MACS), 2019, Japan).

## Data Availability Statement

The raw data supporting the conclusions of this article will be made available by the authors, without undue reservation.

## Author Contributions

MA, FF, and LT conceived and designed the research. MA and MC prepared all the substrates. ML, AM, and ID conducted experiments. ML, FF, AM, and LT wrote the manuscript. SB, AT, AG, SS, and LV revised the manuscript. All authors approved the publication and have read and agreed to the published version of the manuscript.

## Funding

This research was done in the frame of the Viva-Biochar project funded by the Italian Ministry of Agricultural, Food and Forestry Policies (MIPAAF).

## Conflict of Interest

The authors declare that the research was conducted in the absence of any commercial or financial relationships that could be construed as a potential conflict of interest.

## Publisher's Note

All claims expressed in this article are solely those of the authors and do not necessarily represent those of their affiliated organizations, or those of the publisher, the editors and the reviewers. Any product that may be evaluated in this article, or claim that may be made by its manufacturer, is not guaranteed or endorsed by the publisher.

## Supplementary Material

Supplementary Material Linear regression of the Ct values obtained for the different dilution level. The equations of the obtained curves were reported.
