# Supplementary figures and images for: Effects of Biochar on the Growth and Development of Tomato Seedlings and on the Response of Tomato Plants to the Infection of Systemic Viral Agents
Source: Front Microbiol. 2022 May 9;13:862075. doi: 10.3389/fmicb.2022.862075 (PMC9125222; doi:10.3389/fmicb.2022.862075)

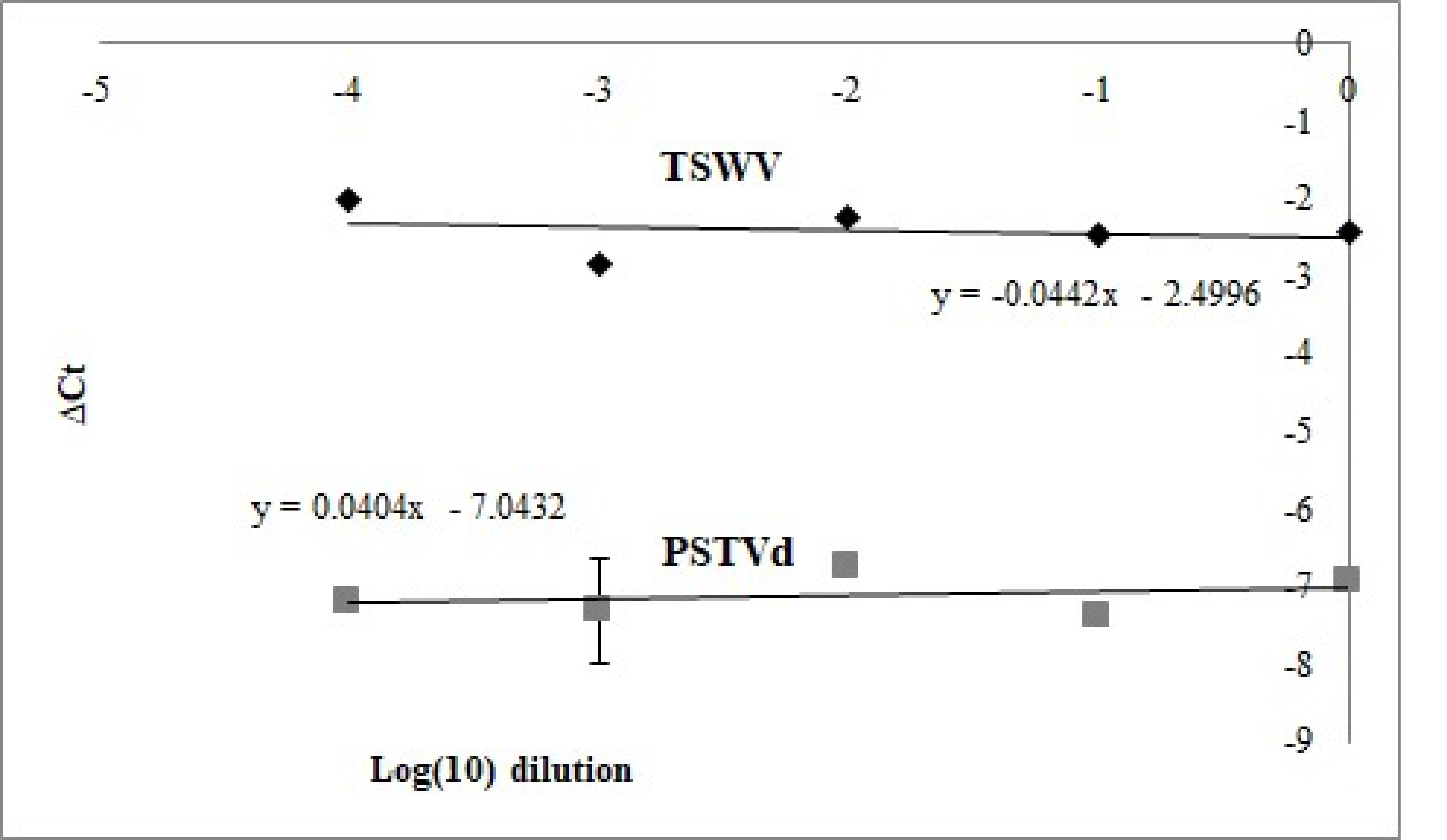

Supplement: Supplementary Material — Linear regression of the Ct values obtained for the different dilution level. The equations of the obtained curves were reported. [file Image_1.TIF]
